# Effect of a Compatibilizer on the Morphology and Properties of Polypropylene/Polyethylentherephthalate Spun Fibers

**DOI:** 10.3390/polym9020047

**Published:** 2017-02-02

**Authors:** Francesco Paolo La Mantia, Manuela Ceraulo, Gaia Giacchi, Maria Chiara Mistretta, Luigi Botta

**Affiliations:** Dipartimento di Ingegneria Civile, Ambientale, Aerospaziale, dei Materiali, Università di Palermo, UdR INSTM di Palermo, Viale delle Scienze, Palermo 90128, Italy; manuela.ceraulo@unipa.it (M.C.); giacchi.gaia@gmail.com (G.G.); mariachiara.mistretta@unipa.it (M.C.M.); luigi.botta@unipa.it (L.B.)

**Keywords:** polyethylentherephthalate, polypropylene, fibers, orientation, compatibilization, particle size

## Abstract

Fibers spun by melt spinning of binary and ternary polypropylene/polyethylenetherephthalate blends have been produced and characterized in order to investigate the effect of a compatibilizer on their morphology and mechanical properties. The compatibilizer was a maleic anhydride-functionalized rubber copolymer. The effect of the compatibilizer was well evident in the isotropic state, as the morphology became very fine, the size of the dispersed particles was very small, and the adhesion was better. The effect of the compatibilizer on the mechanical properties is very relevant, especially in the elongation at break. On the contrary, no relevant effect was observed in the anisotropic oriented fibers. Although the average diameter of the microfibrils of the dispersed phase of the compatibilized blend generated during the hot drawing was much smaller than that of the microfibrils of the same particles of the uncompatibilized blend, the mechanical properties were almost the same. This behavior has been attributed to the length of the smaller microfibrils of the ternary blends, which was lower that of the microfibrils of the binary blend. This has been explained in terms of reduced initial droplet size, and therefore of lesser possibility of stretching the droplets to very long fibrils in these samples.

## 1. Introduction

Blends of different polymers may represent a valid alternative for the production of materials with new characteristics without designing new synthesis that are often complex, expensive, and with a high environmental impact. Therefore, by using proper apparatuses and procedures, it is possible in principle to easily obtain new materials with simpler processes and lower costs than are associated with the preparation of completely new materials. Unfortunately, a key factor in blending different polymers is that in most cases they are immiscible and incompatible; i.e., they display a gross morphology with particles of the dispersed phase poorly adherent to the matrix and poorly distributed into the continuous phase. Consequently, the mechanical properties are poor, and any commercial use is therefore hindered. In these cases, an appropriate compatibilizing system can significantly improve the morphology, and thus the mechanical properties [1,2,3,4,5].

Among the available thermoplastic polymers, the combination of polypropylene (PP) with polyethylentherephthalate (PET) offers many advantages. The tensile strength of PP is high enough for common textile and technical applications; its Young's modulus, however, has a low value that limits its applications. On the other hand, PET is used in many cases for its high resistance and for its high elastic modulus. Mixing the PP with PET is an economical and effective way to improve the properties of PP [6]. Blends PET/ PP present a better resistance to high temperatures than PP alone, while the polyolefin may facilitate the crystallization of PET thanks to a heterogeneous nucleation that further increases the resistance of the blend. Furthermore, the hydrophobic nature of the polyolefin can reduce the sensitivity of PET to humidity, and further facilitate its crystallization. However, because of their different nature, blends of PP/PET are thermodynamically immiscible. A compatibilization of the blends is therefore necessary to avoid obtaining materials with very poor mechanical properties [7,8]. The properties of polymer blend fibers constituted by immiscible polymers strongly depend on the dispersed phase morphology. Many factors can influence the morphology of a polymer blend fiber: nature of the polymers, operating conditions, presence of compatibilizer, type of processing technique, relaxation times of the polymers, etc. Different shapes of dispersed phase—such as sphere, laminar, and fibrillar particles—may form in the matrix in a polymer blend. The production of fibers by melt spinning can result very effectively in fibrillar phase morphology because of the presence of an elongational flow field [7,9,10,11].

Xue et al. [12] investigated the influence of draw ratio, viscosity ratio, and composition ratio of cellulose acetate butyrate/isotactic polypropylenes (CAB/iPP) immiscible blends by scanning electron microscopy (SEM) microscopy. With the increase of the draw ratio, the uniformity of the fibers or elongated spheres in all the iPP samples was increased, and their diameter distributions were narrowed. This indicates that the dispersed iPP phase with larger diameters was more easily deformed than with smaller ones.

Tavanaie et al. [13] studied the effects of the blend ratio on the morphological, rheological and mechanical properties of polypropylene/poly(butylene terephthalate) (PP/PBT) blend fibers using PP-*g*-MAH (polypropylene grafted maleic anhydride) as a compatibilizer. It was observed that for the undrawn samples, the shapes of the PBT dispersed phase changed from fully spherical to fully fibrillar by increasing the PBT dispersed phase content. The elongation at break of the as-spun PP/PBT fiber samples increased with increasing PBT dispersed phase content. 

Marcincin et al. [14] prepared and studied rheological properties, phase structure, and morphology of binary blends of PET/PBT and ternary blends of polypropylene PP/(PET/PBT). A commercial montane (polyester) wax was used as a compatibilizer. The lower viscosity of polyester components and their blends in comparison with PP enables the formation of a blend of PP/polyesters into fibers with highly-deformed particles of dispersed phase into the PP matrix. The higher tensile strength of the fibers with higher content of PBT is probably due to the slightly higher molecular weight of PBT, higher adhesion at interface, and to the stiffening of PBT in the presence of PET component. Furthermore, the positive impact of the compatibilizer on tensile strength has been observed.

Afshari et al. [15] studied the physical properties, morphology, and structure of PP/Nylon6 alloy filaments (10 wt %, 20 wt % Nylon6) made with or without PP-*g*-MAH as compatibilizer. The spun blend filaments were spun and then drawn at different draw ratios. Increasing take-up speed and draw ratio, the values of tenacity and modulus increased, and that of elongation at break decreased for all filaments. The stress–strain curves of the blend filaments show brittle behavior due to the existence of cracks or voids in the matrix (PP) that act as stress concentration points.

Blending PP with PET is an economical and effective way to improve the properties of PP fibers. Moreover, the formation of microfibers as dispersed phase can considerably improve the fibers’ composite Young’s modulus [6].

Kormendy et al. [16] studied the development of phase morphology during the preparation of PP/PET blend fibers as a function of the molecular weight of the PET dispersed phase in the PP matrix. The influence of molecular weight on the morphology of the PET dispersed phase and on the mechanical properties of PP/PET fibers reveals that the tenacity of blend fibers decreases proportionally to the decrease of the molecular weight of the PET component, and the elongation at break increases at the same time.

Jayanarayanan et al. [17] studied some PP/PET (85/15) polymer blends. The micrograph of the blend after extrusion showed the typical incompatible blend morphology, with discrete domains of the minor component dispersed in a continuous phase of the major component. The samples obtained after drawing showed orientation and fibrillation of the PET phase. Large bunches of continuous fibers of PET were observed in the micrographs. 

Mostofi et al. [18] studied the morphology development of binary PP/PET and PP/PET/SEBS (SEBS: styrene ethylene butylene styrene) ternary blends and their fibers by means of scanning electron microscopy (SEM) in conjunction with linear viscoelastic measurements. The SEM micrographs of PP/PET fibers showed well-oriented long microfibrils that became shorter and with larger diameter in the PP/PET/SEBS ternary blend samples. 

Marcinčin et al. [19] prepared fibers from PP/PET blends and analyzed the influence of some non-reactive low-molecular weight compounds. SEM micrographs showed a polyfibrillar structure of the PP/PET blend fibers with long microfibrils of PET in the PP matrix that result from the interaction at the interface.

Fakirov et al. [20] investigated the contribution of coalescence to microfibril formation in cold drawn fibers of polymer blends of PP and PET with and without a compatibilizer. The compatibilized blends showed completely different results: their microfibrils were very short in length with respect to the uncompatibilized blend. The amount of the added compatibilizer was sufficient to coat the PET particles, preventing the coalescence.

Si et al. [6] investigated some PP/PET blends with PP-*g*-AA (polypropylene-graft-acrylic acid) as a compatibilizer. The PET islands could be deformed into micro-fibers after hot stretching if there was a good interaction between the PP and PET surfaces. The results showed that the addition of PP-g-AA improved the mechanical properties of the fibers with respect to the PP matrix, but this improvement was higher at low compatibilizer content. No comparison was made with the uncompatibilized blend.

Friedrich et al. [21] prepared blends utilizing recycled PET, PP, and E-GMA (ethylene-*co*-glycidyl methacrylate) as compatibilizer. The blends were extruded and then cold drawn. The micrographs of the cryogenic fracture surfaces of the extruded compatibilized samples showed a rather homogeneous and isotropic dispersion of PET spheres in the PP matrix. The PET particles in this blend were two to three times smaller than those in the blend without compatibilizer. After drawing, the blend components were transformed into a highly-oriented state. The SEM micrographs show very well-orientated PET fibrils with a high aspect ratio. It can be seen that the fibrils obtained in the blends without compatibilizer were longer (almost no ends could be seen) than those obtained in blends with compatibilizer. This can be explained by the influence of E-GMA, which covers the PET particles during mixing and prevents their coalescence during drawing. This highly fibrillar structure also reflects the mechanical properties of the drawn blends. The tensile modulus of the drawn bristles was two to four times higher than the modulus of the bristles after extrusion. A similar dependence could be observed for the tensile strength. Surprisingly, the samples with the compatibilizer exhibited lower values under these testing conditions. This can be explained by the influence of the compatibilizer, which acts as a softening agent (see lower values in case of the extruded samples). In addition, its presence in the blends leads to a shorter length of the microfibrils (and therefore reflects the typical aspect ratio effect in the drawn blends), as was mentioned above. Additionally, in the paper of Yi et al. [22], the presence of a compatibilizer—an epoxy functionalized polypropylene—made the microfibrillation of the dispersed PET particles in a blend with PP less effective.

The aim of this work was to study the effect of a rubbery compatibilizer on the morphology and mechanical properties of melt spun PP/PET fibers at different hot draw ratios. The morphology and mechanical properties indicated that the effect of the compatibilizer is to depress the fibrillation of the PET dispersed phase. This was attributed mainly to the lower value of the size of the PET particles that make the fibrillation due to the elongational flow less effective.

## 2. Materials and Methods

The materials used in this work were a sample of polypropylene (Capilene®E50E, Carmel Olefins Ltd., Haifa, Israel) with a melt flow index of 1.8 at (230 °C/2.16 Kg), and PET (Cleartuf P82, M&G Polimeri Italia, Alessandria, Italy) having intrinsic viscosity 0.82 dL/g and *T*_m_ = 249 °C. The polymer used as compatibilizer was Kraton FG 1901X which is a SEBS (Styrene Ethylene Butylene Styrene) copolymer grafted with maleic anhydride. 

The blends were prepared by extrusion in a counter-rotating twin-screw compounder (Brabender), D = 45 mm, L/D = 7, with a temperature profile of 180-240-270-270 °C and at a screw speed of 60 rpm. The extrusion temperatures were 270 °C for all blends. Before blending, all samples were dried in a vacuum oven at 120 °C overnight. The blends compositions and sample codes of all the investigated materials are reported in Table 1.

The fibers were spun by using the drawing module of a capillary viscometer (Rheologic 1000, CEAST, Torino, Italy) at 270 °C for all the blends and at 20 °C for pure PP (to be described in more detail later).

Rheological measurements in shear flow were carried out using a plate–plate MARS III rheometer (Thermofisher, Waltham, MA, USA) at 270 °C, in the frequency range 0.1–400 rad/s. Tests were conducted on compression-molded samples (*D* = 25 mm) obtained using a Carver (Wabash, IN, USA) laboratory press set at 270 °C for all samples, compression time 3 min, pressure about 20 MPa. The tests were conducted with a strain of 5%, chosen after performing a standard procedure based on strain sweep tests in order to determine the limits of the linear viscoelastic regime. Reproducibility was about ± 5%.

Rheological measurements in shear flow and in non-isothermal elongational flow were performed in a capillary rheometer (Rheologic 1000, CEAST, Torino, Italy) equipped with a tensile module at 270 °C for PP, PET, and blends. Capillary diameter was 1 mm, and the length-to-diameter ratio was 40; the range of shear rates was chosen based on the typical shear rates applied during industrial extrusion operations. The extruded filament passes through a pulley system and is then drawn by two counter-rotating rolls. The run is carried out by pulling the filament—extruded at a given flow rate—at a rotational speed which increases with a linear acceleration of 100 rpm s^−1^. The test ends with the breaking of the filament. The force measured in the molten filament at breaking corresponds to the “melt strength” (MS), while the ratio between the drawing speed at breaking (measured on the basis of the rotating speed of the pulley at the moment when the filament breaks), and the extrusion velocity (in runs in which the drawing velocity increases with a constant acceleration) corresponds to the breaking stretching ratio (BSR), which is practically the maximum elongation that the molten polymer can bear under those specific experimental conditions. Therefore, characterization through MS and BSR measurement was performed simultaneously with the fiber production. Reproducibility of the MS and BSR results was satisfactory (± 7%).

As already pointed out, during the non-isothermal elongational tests, the apparatus allows fibers to be prepared and collected at different draw ratios. The extrusion apparent shear rate was 60 s^−1^. The hot draw ratio, *DR*_h_, adopted for all materials was 10. *DR*_h_ was calculated as:

*DR*_h_ = *D*_0_^2^/*D*_f_^2^ where *D*_0_ is the diameter of the capillary, and *D*_f_ is the diameter of the fibers. 

FT-IR spectra were collected by a Spectrum One spectrometer by Perkin-Elmer (Waltham, MA, USA), using Spectrum One software. Spectra were measured with eight scans and a 4 cm^−1^ resolution. To quantitatively compare the spectra of the two blends, we considered a normalization over the vibration band of –CH_2_– groups (1462 cm^−1^).

Tensile tests were performed using an Instron (High Wycombe, UK) mod. 3365 universal machine on samples (90 × 10 × 0.5 mm^3^) cut off the compression-molded sheets. The elastic modulus (*E*) was measured at a speed of 1 mm/min. When the deformation was about 10%, the crosshead speed was increased to 100 mm/min until break. The values for *E*, tensile strength (*TS*), and elongation at break (*EB*) were calculated as the average of 10 tests.

The morphology of all the extruded and compression-molded samples was evaluated via scanning electron microscopy Phenom Pro X (Phenomworld, Eindhoven, The Netherlands) SEM micrographs were obtained on samples fractured in liquid nitrogen and sputtered with gold to make them conductive. For the extruded samples, the SEM micrographs refer to the plane perpendicular to the extrusion direction. To better observe the dispersed phase of the fibers, the matrix was etched by boiled xylene in a Soxhlet apparatus.

## 3. Results

### 3.1. Compatibilization

**Figure 1:**
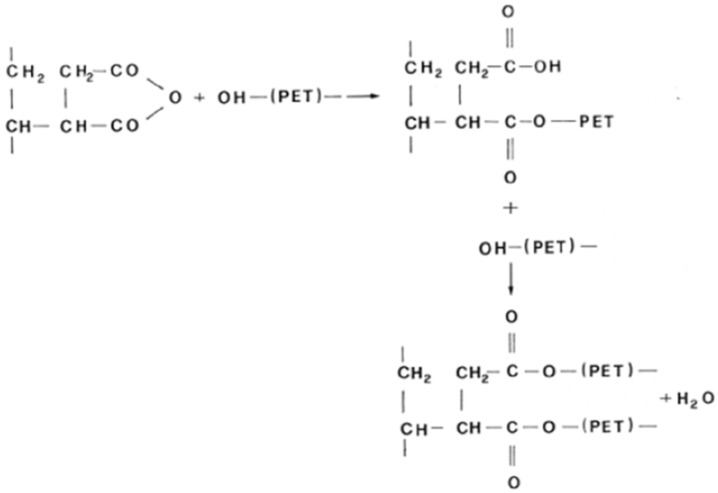
Scheme of the reactions between SEBS-*g*-MA (styrene ethylene butylene styrene grafted with maleic anhydride) and PET

Fourier transform infrared spectrometry (FT-IR) was used to confirm that the reaction between PET and the maleic anhydride took place—according to the reactions reported in Figure 1—during the extrusion in the presence of the compatibilizer.

This possible reaction mechanism between the maleic ring and the terminal group –OH of the PET shown in Figure 1 occurs in two steps. After the first step, there will be a new copolymer with an ester group and a carboxylic acid. However, at the end of the last step, a copolymer with two ester groups will be formed. The copolymers formed during this process act as a bridge between the two phases, improving adhesion of the two polymers and the properties of the blend [8].

**Figure 2:**
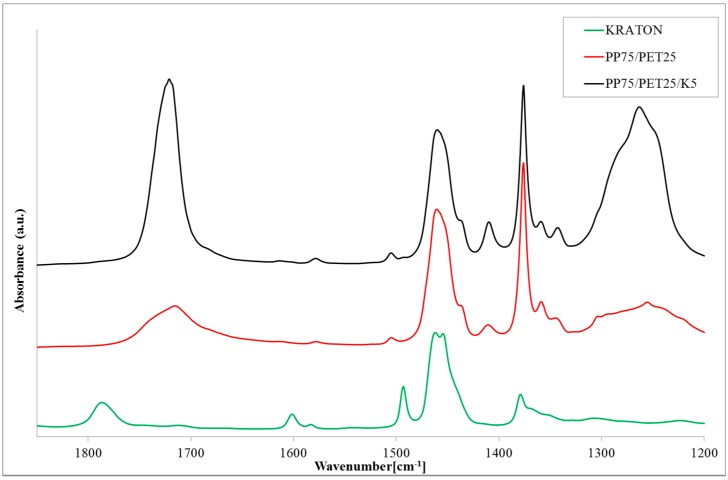
Fourier transform infrared (FT-IR) spectra of compatibilizer used in this work (K, Kraton), binary blend (PP75/PET25), and compatibilized blend (PP75/PET25/K5).

FT-IR spectra of the copolymer with the grafted maleic anhydride (Kraton), PP75/PET25, and PP75/PET25/K5 are shown in Figure 2.

The presence of maleic anhydride (MA) in the compatibilizer is confirmed by the peak at 1789 cm^−1^. This peak is absent in the spectrum of the compatibilized blends, confirming that the reaction takes place during the extrusion. The compatibilized blend shows the peaks of the carboxylic acids (1720 cm^−1^) and of the ester groups (1265 cm^−1^) higher than those of the binary blend due to the presence of the PET dispersed phase establishing that new groups are formed during the extrusion.

**Figure 3:**
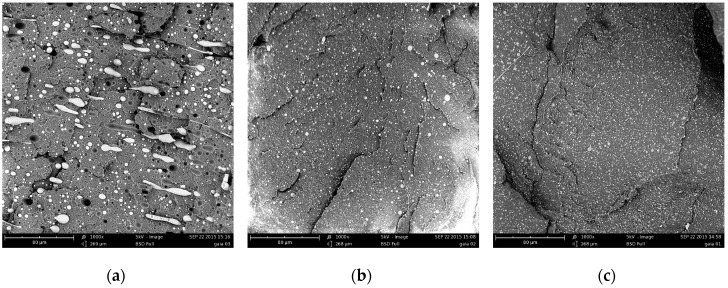
SEM micrographs of the cryogenically fractured extruded samples: (**a**) PP75/PET25; (**b**) PP75/PET25/K2.5; and (**c**) PP75/PET25/K5.

SEM images of the extruded blends are shown in Figure 3.

The incompatibility of the two polymers is confirmed by the morphology of the binary blend as revealed by the SEM micrograph of Figure 3a. The continuous phase is composed of polypropylene, and the discrete particles of PET are large and with a wide particle size distribution. The morphology of the blend is remarkably modified by adding small amounts of Kraton. The micrographs of the blends with 2.5% and 5% of compatibilizer are reported in Figure 3b,c, respectively. Contrary to Figure 3a (uncompatibilized blend), the images of the compatibilized blends demonstrate that the two phases adhere very well, even at low Kraton content. Furthermore, the dimensions of the discrete particles are very small.

These features—and in particular the reduction of the dimensions of the PET particles—are only slightly improved by increasing the content of Kraton. It is interesting to note that the shape of the dispersed particles is quite elongated, and this is not observed in the compatibilized blends. The elongation can be due to the action of the convergent flow at the end of the extrusion head. This presumably does not occur in the compatibilized blends because of the very small size of the particles that cannot be elongated during the elongation.

### 3.2. Rheological Characterization

**Figure 4:**
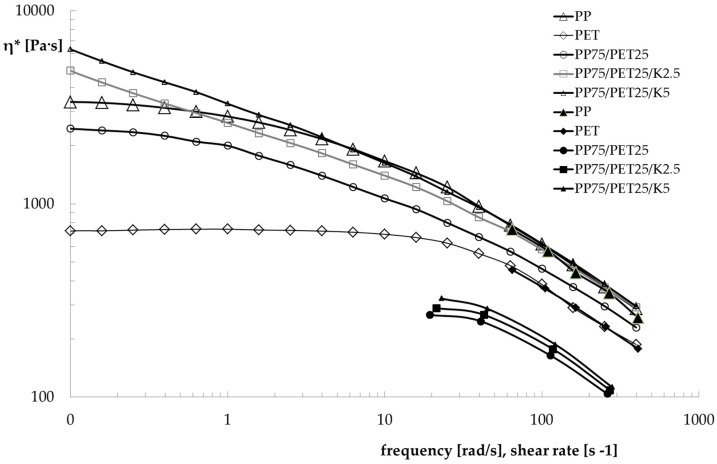
Flow curves of the investigated samples. The empty symbols refer to rotational rheometer (frequency rad/s x axis), and the full symbols to the capillary viscometer (shear rate s^−1^ x axis).

The flow curves of all blends measured with both the rotational rheometer and the capillary viscometer are reported in Figure 4.

**Figure 5:**
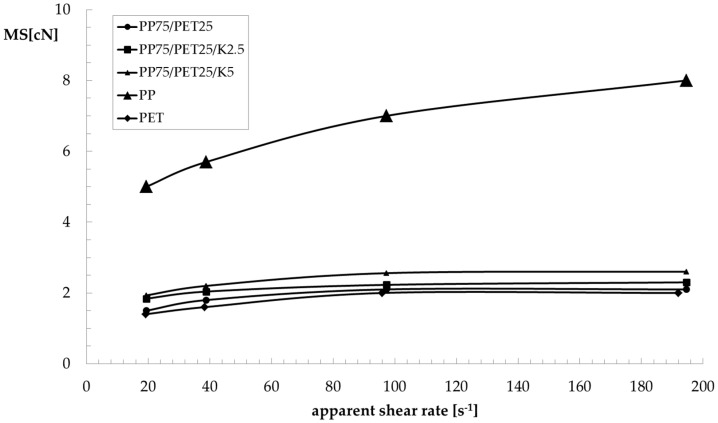
Melt strength (MS) as a function of the apparent shear rate of the PP/PET blends.

**Figure 6:**
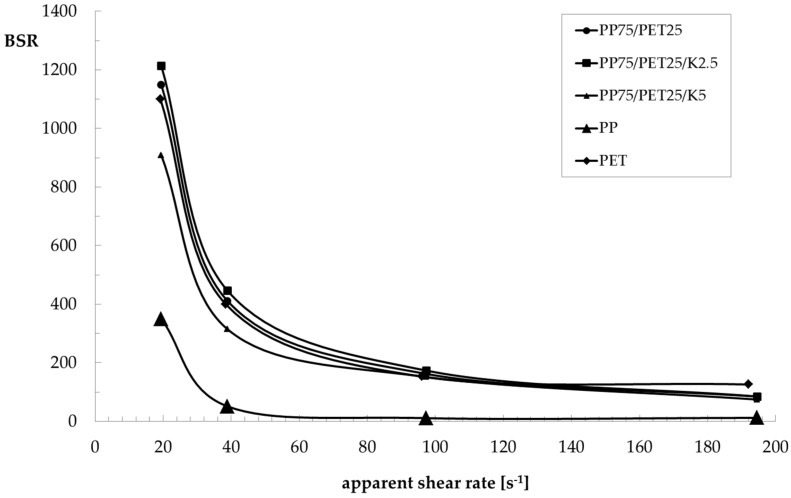
Breaking stretching ratio (BSR) as a function of the apparent shear rate of the PP/PET blends.

As can be observed, the PET sample shows a typical Newtonian behavior in a wide frequency region, and the viscosity only decreased at high values of frequency/shear rate. On the contrary, the PP shows a Newtonian plateau even in the low-frequency region. The PP sample shows values of complex viscosity much higher than the PET sample. Moreover, the non-Newtonian behaviour is much more pronounced. The viscosity of the uncompatibilized blend is lower than that of the PP matrix, and with a similar non-Newtonian behaviour. Due to its composition, the viscosity is dominated by the behaviour of the matrix. The presence of the compatibilizer strongly modifies this behaviour. Indeed, not only the viscosity increases (even more than the matrix), but the Newtonian plateau also disappears and the non-Newtonian behaviour becomes more pronounced with increasing Kraton content. Both behaviours are certainly a demonstration of the better adhesion between the two phases and of the lower dimensions of the dispersed particles, which increase the contact surface between matrix and dispersed particles. As can be observed in Figure 4, the flow curves obtained in rotational and capillary flow of the two components superimpose as predicted by the Cox–Merz rule, while this does not hold true for the blends. This behaviour has already been observed [23,24,25,26] and correlated with the convergent flow at the inlet of the capillary that orients the dispersed particles along the flow direction, reducing the resistance to the flow. In Figure 5 and Figure 6, the melt strength (MS) and the breaking stretching ratio (BSR) values, respectively, for the components and the three blends are reported as a function of the apparent shear rate in order to evaluate the spinnability of these blends in comparison with that of the two components. As expected, the MS values increase with the shear rate while the BSR values decrease for all samples. PP shows higher values of melt strength than PET, while the opposite is true for the BSR. The MS and BSR values of all the blends are intermediate, between those of the pure components. The binary blend shows values of MS only slightly lower than those of the blend with 2.5% of Kraton. The effect of the compatibilizer is more evident for the blend with 5% Kraton, which shows slightly higher MS values and lower BSR values. As for the melt strength, this effect is clearly due to the increase of the viscosity in the presence of the compatibilizer, while the improved stretchability is due to the better homogeneity of the compatibilized blends. Finally, the decrease of the BSR with increasing Kraton content can be due to the higher viscosity of this blend. It is worth noting that all of the blends show a spinnability intermediate between those of the two components, and this spinnability is slightly better for the compatibilized blend. In fact, the BSR values are similar for all the blends.

### 3.3. Mechanical and Morphological Characterization

The mechanical properties of the isotropic sheets prepared from the pure polymers and from the three blends are reported in Table 2.

Pure PET exhibits the highest value of the Young modulus and a low value of elongation at break, especially in comparison with PP. The elastic modulus of the binary blend is intermediate between the values of the pure polymers, while the tensile strength and elongation at break are very low—lower than those of both components. This behavior and the brittleness shown by this blend clearly evidence the strong incompatibility between the two phases, as well as the lack of adhesion. The presence of the compatibilizer strongly changes this behavior. Indeed, both tensile strength and elongation at break increases, the blends become ductile, and both tensile strength and elongation at break are between those of the two components. In presence of 5% Kraton, the deformability of the blend becomes very high. On the contrary, the elastic modulus surprisingly decreases with increasing amount of compatibilizer, and the tensile strength remains lower than those of both components. This can be interpreted considering the rubbery nature and the very low value of elastic modulus and tensile strength of the compatibilizer. The relevant increase of the elongation at break in the presence of 5% Kraton with respect to the rise in the presence of 2.5% could also be due to the same reason and to the better adhesion between the two phases.

**Figure 7:**
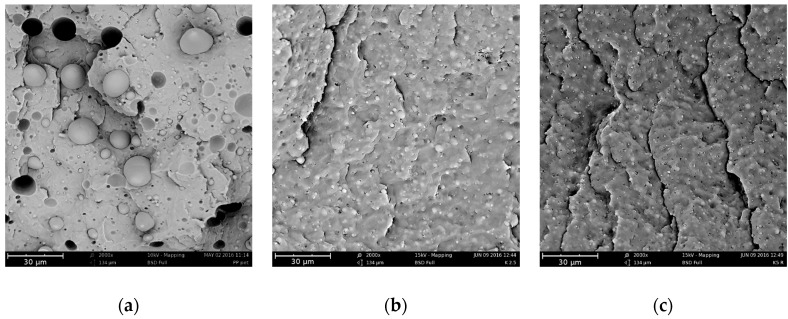
SEM micrographs of the cryogenically fractured isotropic sheets: (**a**) PP75/PET25; (**b**) PP75/PET25/K2.5; and (**c**) PP75/PET25/K5.

The SEM micrographs of the sheets of the three blends are reported in Figure 7. The morphology of the isotropic sheets is similar to that of the extruded samples: lack of adhesion, badly dispersed large PET particles in the uncompatibilized blend, and smaller particles and better adhesion in the two compatibilized blends. However, some difference can be noticed in the uncompatibilized blend. Indeed, the particles are spherical and with larger dimensions. This is presumably due to coalescence phenomena during the preparation of the sheets by compression molding. This phenomenon is prevented in the compatibilized blends by the presence of the Kraton or of the copolymer formed by Kraton and PET that reduces the interfacial tension and covers the dispersed particles. 

**Figure 8:**
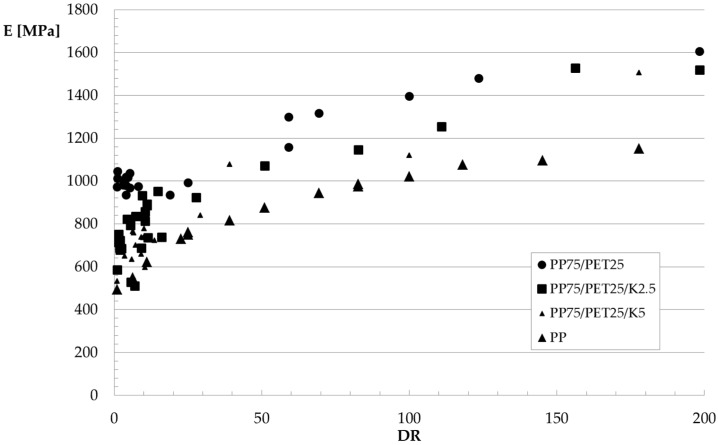
Elastic modulus as a function of the hot draw ratio.

**Figure 9:**
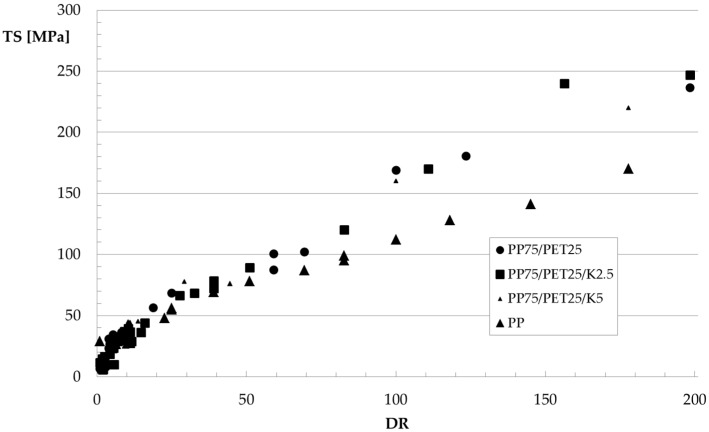
Tensile strength as a function of the hot draw ratio.

**Figure 10:**
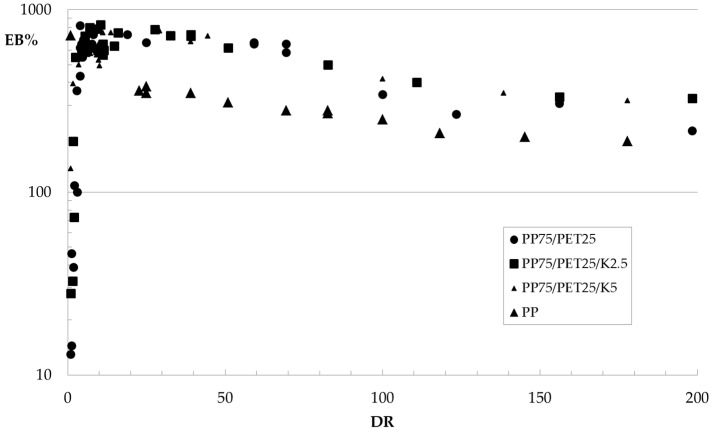
Elongation at break as a function of the hot draw ratio.

The values of the elastic modulus *E*, tensile strength *TS*, and elongation at break *EB* of the fibers are reported in Figure 8, Figure 9 and Figure 10, respectively, as a function of the hot draw ratio (*DR*). For comparison, the experimental data for the fibers of the pure PP matrix are also reported. All of the collected data have been reported in the Figures.

**Figure 11:**
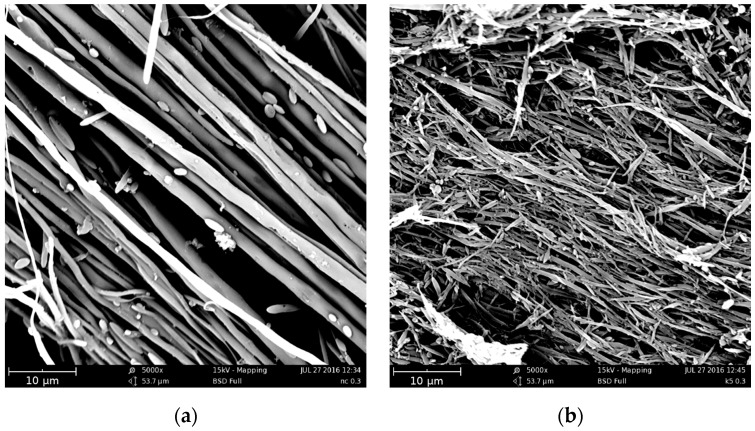
SEM micrographs of PET fibrils coming from fibers with draw ratio (*DR)* of about 100: (**a**) PP75/PET25 blend; (**b**) PP75/PET25/K5 blend.

Elastic modulus and tensile strength increase with the hot draw ratio because of the orientation achieved by the macromolecules due to the applied elongational flow. As for the fibers of the blends, an unexpected behavior can be observed for the elongation at break. Indeed, as expected, while the elongation at break–draw ratio curve of the PP fibers decreases, with the orientation, at low values of the draw ratio, the elongation at break of the fibers of the blends dramatically increases with *DR*, while it decreases with further increasing *DR*. The fibers of the non-compatibilized blend and of the ternary blend with 2.5% Kraton become ductile, reaching very high values of the elongation at break with the orientation, so a brittle–ductile transition after the application of the elongational flow can be observed. This behavior has been already found for some amorphous polymers [27] and blends [28]. In [27], this behavior has been interpreted considering that during the tensile test, the dispersed particles—elongated like the macromolecules of the matrix because of the flow—do not act as a defect in the blend like in the isotropic samples, because of the larger contact surface that mitigates the effect of the poor adhesion. The increase of the contact area can therefore be considered responsible for the increased ductility of the fibers. SEM micrographs of PET fibrils coming from fibers with a *DR* of about 100 are reported in Figure 11a,b. The elongational flow deforms the spherical or ellipsoidal particles (see Figure 3) into fibrils, confirming the increase of the contact area between the matrix and the dispersed particles.

As for the effect of the compatibilizer, no remarkable difference can be observed in any of the investigated mechanical properties, as the increase of elastic modulus and tensile strength or the increase/decrease of the elongation at break are very similar for the three systems. Despite the better adhesion, the final properties of the oriented systems are very similar for the fibers made from binary and ternary blends. 

Considering the SEM micrographs reported in Figure 11, in both cases, the PET phase is drawn into fibrils by the elongational flow, but some smaller deformed particles are also present in the blends. In particular, the binary blend shows very long fibrils of PET with diameters of about 1–2 μm. The small particles of PET are probably formed after a rupture of some very long and thin fibril. While the PET droplets form long microfibrils in the PP/PET blend, in the PP/PET/Kraton blend, the minor phase is drawn into shorter fibrils having diameters even smaller than 1 μm. Despite the different type of compatibilizer, the different morphologies achieved between the compatibilized and uncompatibilized blends described above are in full agreement with that found in [18,19,20]. In accordance with [18,19,20], the lower length of the fibrils observed in the compatibilized fibers can be explained by the reduced initial droplet size (see Figure 3), and therefore by the reduced possibility of stretching the droplets to very long fibrils in these samples. Moreover [20], the presence of the compatibilizer hinders the coalescence of the droplets. The lower size of the PET particles in the compatibilized blends induces a lower diameter, but also a much lower length of the microfibrils during the hot drawing.

It is well known that the length-to-diameter ratio of the reinforcing fibrils is a key parameter to improve the mechanical properties of the matrix. However, no mechanical data have been presented on these systems in the previous papers, so the different type of morphology has not been correlated with the mechanical properties. The lower length-to-diameter ratio of the microfibrils does not allow for any significant improvement of the mechanical properties of the fibers of the compatibilized blends with respect to those of the fibers of the uncompatibilized blend. 

The effect of the compatibilizer is then different considering isotropic unoriented sheets and anisotropic oriented fibers. Indeed, in the case of isotropic specimens, the reinforcing action is enhanced with decreasing size of the particles, and in this case, the presence of the compatibilizer plays a beneficial role. On the contrary, this same effect is negative in the anisotropic specimens, as the dispersed particles give rise to reinforcing fibrils that are too short to improve the mechanical properties of the matrix.

A last comment can be made about the comparison with the behavior of the fibers made of pure PP. The effect of the orientation dramatically changes the behavior observed for the unoriented samples (see Table 2) with respect to the blends. Indeed, the elastic modulus and tensile strength of the fibers of the blends become higher than that of the PP matrix, and this is interpreted in terms of better reinforcing effect of the oriented fibrils of the high-modulus PET with respect to the spherical particles of the isotropic sheets. The comparison of the elongation at break is much more complex. Indeed, the PP fibers show the typical decrease of the deformability with the draw ratio because of the orientation of the macromolecules along the drawing direction, while the same behavior can be observed for the fibers of the blends only after the brittle-to-ductile transition.

## 4. Conclusions

A blend made of PP as a matrix with a PET dispersed phase has been compatibilized with a rubbery sample of SEBS copolymer grafted with maleic anhydride. The compatibilization improves the viscosity in shear flow of the uncompatibilized blend, and significantly improves the rheology of the blend in non-isothermal elongational flow. The improved and more refined morphology of the compatibilized blend strongly influences the mechanical behavior of the isotropic sheets of the binary blend; in particular, the elongation at break that dramatically increases, while the modulus and tensile strength are reduced because of the presence of the rubbery phase. The anisotropic fibers obtained by melt spinning show a different behavior. Indeed, the presence of the compatibilizer does not give rise to any significant difference in the mechanical properties with respect to those of the binary blend. This has been mainly attributed to the smaller diameter of the droplets of the dispersed phase of the ternary blend, which gives rise to shorter fibers. Finally, a brittle–ductile transition at low values of the draw ratio has been observed for the blends.

## Figures and Tables

**Table 1:** Composition of the investigated samples.

Blends	PP (wt %)	PET (wt %)	Kraton (phr)	Sample code
PET	-	100	-	PET
PP	100	-	-	PP
PP/PET	75	25	-	PP75/PET25
PP/PET/Kraton	75	25	2.5	PP75/PET25/K2.5
PP/PET/Kraton	75	25	5	PP75/PET25/K5

PP: polypropylene; PET: polyethylenterephthalate

**Table 2:** Mechanical properties of isotropic samples.

PP/PET/Kraton	Elastic modulus (MPa)	Tensile strength (MPa)	Elongation at break (%)
0/100/0	1208 ± 7%	18.7 ± 6%	22 ± 6%
100/0/0	493 ± 8%	29.1 ± 4%	724 ± 6%
75/25/0	973 ± 9%	6.7 ± 8%	11 ± 14%
75/25/2.5	572 ± 6%	11.7 ± 4%	28 ± 6%
75/25/5	526 ± 4%	11.5 ± 3%	130 ± 5%
